# Wearable sensors for clinical applications in epilepsy, Parkinson’s disease, and stroke: a mixed-methods systematic review

**DOI:** 10.1007/s00415-018-8786-y

**Published:** 2018-02-09

**Authors:** Dongni Johansson, Kristina Malmgren, Margit Alt Murphy

**Affiliations:** Department of Clinical Neuroscience, Institute of Neuroscience and Physiology, Sahlgrenska Academy, University of Gothenburg, Gothenburg, Sweden

**Keywords:** Wearable sensors, Parkinson’s disease, Epilepsy, Stroke, Systematic review

## Abstract

**Objectives:**

Wearable technology is increasingly used to monitor neurological disorders. The purpose of this systematic review was to synthesize knowledge from quantitative and qualitative clinical researches using wearable sensors in epilepsy, Parkinson’s disease (PD), and stroke.

**Methods:**

A systematic literature search was conducted in PubMed and Scopus spanning from 1995 to January 2017. A synthesis of the main findings, reported adherence to wearables and missing data from quantitative studies, is provided. Clinimetric properties of measures derived from wearables in laboratory, free activities in hospital, and free-living environment were also evaluated. Qualitative thematic synthesis was conducted to explore user experiences and acceptance of wearables.

**Results:**

In total, 56 studies (50 reporting quantitative and 6 reporting qualitative data) were included for data extraction and synthesis. Among studies reporting quantitative data, 5 were in epilepsy, 21 PD, and 24 studies in stroke. In epilepsy, wearables are used to detect and differentiate seizures in hospital settings. In PD, the focus is on quantification of cardinal motor symptoms and medication-evoked adverse symptoms in both laboratory and free-living environment. In stroke upper extremity activity, walking and physical activity have been studied in laboratory and during free activities. Three analytic themes emerged from thematic synthesis of studies reporting qualitative data: acceptable integration in daily life, lack of confidence in technology, and the need to consider individualization.

**Conclusions:**

Wearables may provide information of clinical features of interest in epilepsy, PD and stroke, but knowledge regarding the clinical utility for supporting clinical decision making remains to be established.

**Electronic supplementary material:**

The online version of this article (10.1007/s00415-018-8786-y) contains supplementary material, which is available to authorized users.

## Introduction

Wearables is the common term for devices integrated in garments or designed as wearable accessories. Wearables with built-in sensors such as accelerometers, gyroscopes, and magnetometers allow continuous long-term monitoring of movement patterns or physiological variables. In neurology, wearables offer new possibilities to achieve continuous and objective symptom monitoring in clinical as well as out-of-hospital settings. Parkinson’s disease (PD) and stroke are the two neurological conditions, where accelerometry-based technology has been applied most [1]. There is also a growing interest in using wearable devices to detect seizures in epilepsy [2]. Although accelerometry-based devices were introduced for measuring physical activity already in the 1980s and the necessary data management technology has been available since the 1990s, it is only recently that the use of wearable accelerometry-based devices has started to take hold in clinical applications. With increasing use in different neurological diseases, it is necessary to evaluate the clinical efficacy and usefulness of measures derived from wearables. It is also necessary to identify common barriers and facilitators for clinical applications. The different needs for monitoring in the diseases addressed in this review create specific challenges for the use of wearables, but there are also several general problems, where solutions from one disease area might be generalizable and of interest to the other. Individuals with a neurological condition might find it difficult to interact with technology due to physical or cognitive limitations, and visually conspicuous wearables may increase disease stigmatization [3]. A comprehensive understanding and evaluation of technology and end-user preferences is important to further facilitate integration of wearables into clinical practice.

The purpose of this systematic review was to provide an overview and to aggregate both quantitative and qualitative knowledge from clinical research with wearable sensor technology in individuals with epilepsy, PD, and stroke. Clinical application areas, main findings, and clinimetric properties of measures derived from wearables, proportion of reported missing data, and adherence along with perceived experiences and preferences of wearables will be summarized for all three diseases.

## Methods

A systematic literature search was performed to identify the most relevant quantitative and qualitative studies. Search strategies were created based on the PICO framework (Population, Intervention, Comparison, and Outcome) [4]. The SPIDER tool (Sample, Phenomenon of Interest, Design, Evaluation, and Research type) was used as an extra search strategy to identify qualitative studies [5]. MeSH terms and free keywords were used for searches in PubMed, Scopus, Ovid SP, CINHAL, and Cochrane Library Databases. The search results from different databases were largely overlapping, but PubMed showed the best coverage for quantitative and Scopus for qualitative studies in terms of relevance and number of articles. Therefore, quantitative studies were selected from PubMed and qualitative studies from Scopus. The searches were limited to articles in English published between 1995 and 2015, and updated in January 2017 (see search strategies in Supplementary information 1).

The inclusion criteria for studies reporting quantitative data were: (1) peer-reviewed original studies; (2) use of wearable sensors (such as accelerometers, gyroscopes, and magnetic sensors) in people with epilepsy, PD, or stroke; (3) monitoring of movements and physiological signs; and (4) study outcomes related to symptoms or impairments with clinical relevance to epilepsy, PD, or stroke. The exclusion criteria were: (1) less than ten participants; (2) conference proceedings, reviews, case reports, non-human studies, and grey literature (e.g., theses, reports, policy and government documents, and study protocols); and (3) implantable sensors.

The inclusion criteria for studies reporting qualitative data were: (1) peer-reviewed original studies; (2) analysis of primary qualitative data; and (3) studies on patients’ or clinicians’ experiences and/or preferences on acceptability, expectations, feasibility, and/or usability of using wearables. Studies were excluded if the qualitative data analysis was not related to wearables.

**Fig. 1:**
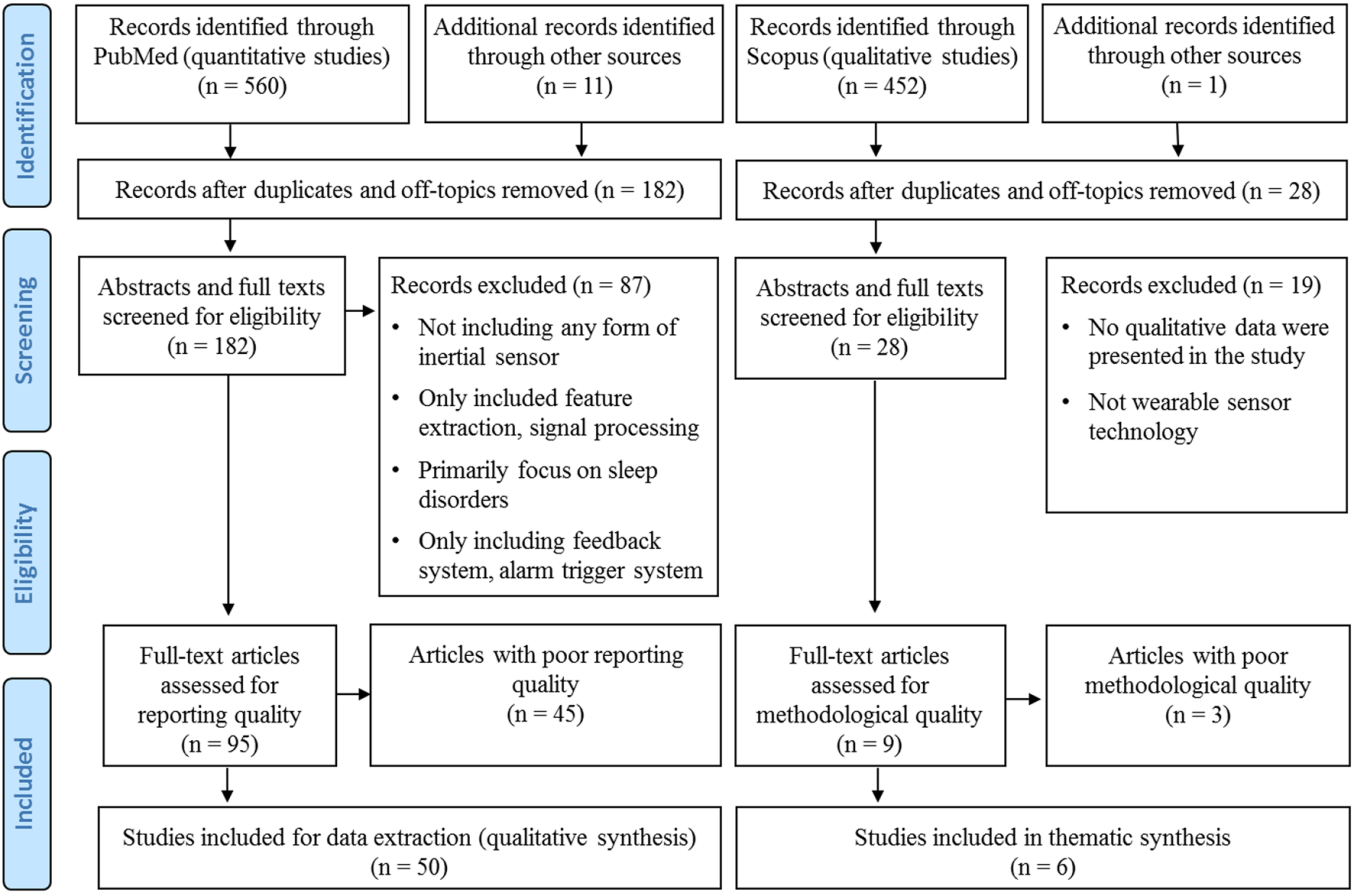
Flow diagram of the systematic review selection process

Each title and abstract was screened for inclusion by two independent reviewers (DJ, MAM). Discrepancies were resolved by discussions between the two reviewers until a consensus was reached. Relevant literature known to the authors from other sources was also screened for inclusion. Reference lists of all included studies were searched manually to identify additional studies (Fig. 1).

### Quality assessment

A critical appraisal of the reporting quality of the quantitative studies eligible for the review was performed using the Strengthening the Reporting of Observational Studies in Epidemiology (STROBE) statement [6]. The STROBE was developed to improve the reporting quality of observational studies and to facilitate critical appraisal and interpretation of the study results [7]. The reporting quality is an essential element for a study, and indispensable for proper appraisal of internal and external validity of findings [8]. The STROBE checklist and particularly the key components of the STROBE, coherent with the basic requirements to quality assessment of observational studies were used. Thus, in the current review, all 22 items of the STROBE were assessed, and sufficient reporting quality was assigned to studies which met the following standards: a clear statement of objective(s) (item 3), described eligibility (inclusion and exclusion) criteria (item 6), defined outcome variables (items 7 and 11), described statistical methods used (item 12), description of number and characteristics of participants provided (items 13 and 14), outcomes measures and main results (items 15 and 16) and provided summary and interpretation of key results in concurrence with the study aims provided (items 18, 20, and 21). Fulfilment of these 12 STROBE items corresponds to more than 50% of all 22 STROBE items, a cutoff which has been used in several previous studies [9–11]. All 22 items of the STROBE statement were discussed between two reviewers before quality assessment (DJ, MAM) to reach a consensus of understanding on each item of the checklist. The first 20 articles from an alphabetically sorted list were scored independently by the two reviewers to ensure consensus. The rest of the included articles (*n* = 73) were then scored by one reviewer (DJ), and any uncertainties were discussed and rescreened with the second reviewer (MAM).

The methodological quality of studies that reported qualitative data was assessed with the Critical Appraisal Skills Programme (CASP) [12]. The questions of the CASP targeting aims, methodology, design, recruitment, data collection, data analysis, ethical considerations, and findings needed to be fulfilled (see supplementary information 2).

### Data extraction and synthesis

The aim, sample characteristics, main findings, proportion of reported missing data, and adherence were extracted from studies with sufficient reporting quality only. A thematic analysis was used to synthesize all text from the results sections reporting qualitative data of the studies that passed the critical appraisal checklist. A free line-by-line coding was performed using the Nvivo software (QSR International, Melbourne, Australia, version 11.0) [13]. Descriptive themes and subthemes were then constructed based on the free codes. Analytical themes were generated and developed in relation with the descriptive themes.

## Results

The initial PubMed and Scopus literature search resulted in the retrieval of a total of 1012 articles (Fig. 1). From these, 210 studies were included in the full-text review, and 104 studies were eligible for quality assessment. Fifty quantitative studies were assigned sufficient reporting quality and 6 out of 9 studies that reported qualitative data passed the critical appraisal for methodological quality. Thus, 50 studies reporting quantitative data and 6 studies reporting qualitative data were included for further data extraction and synthesis (Fig. 1). Of the 50 papers reporting quantitative data, 5 (10%) were in epilepsy, 21 (42%) in PD, and 24 (48%) in stroke. All studies in epilepsy were conducted in a hospital environment. In PD, 13 studies were conducted in a laboratory, one study in a hospital environment, and 7 studies in a free-living environment. In stroke, 4 studies were conducted in a laboratory, 6 in a hospital environment, and 14 studies used wearables in a free-living environment. Qualitative data were reported in one study in epilepsy, three in PD, and two in stroke. A meta-analysis was considered unfeasible for the quantitative studies due to large variation of study aims and designs.

### Studies reporting quantitative data

An overview of clinical application areas, population characteristics along with methods, and the main findings is provided in Supplementary Table 1. In epilepsy, wrist-worn sensors with built-in accelerometers were used for detection and classification of seizures in hospital settings [14–18]. In PD, wearables were used to detect and quantify cardinal motor symptoms including bradykinesia [19], tremor [20, 21], and postural sway [22, 23] as well as medication-evoked adverse symptoms such as dyskinesia [24–27] and motor fluctuations [28, 29]. Wearables were also used to quantify sleep disturbances [30], gait measures [31, 32], freezing of gait [33, 34], missteps and fall [35, 36], and physical activity levels [37–39]. In stroke, upper extremity activity [40–51], walking, and physical activity levels were investigated in several studies using step and activity counts [52–62].

### Wearables in laboratory environment

**Fig. 2:**
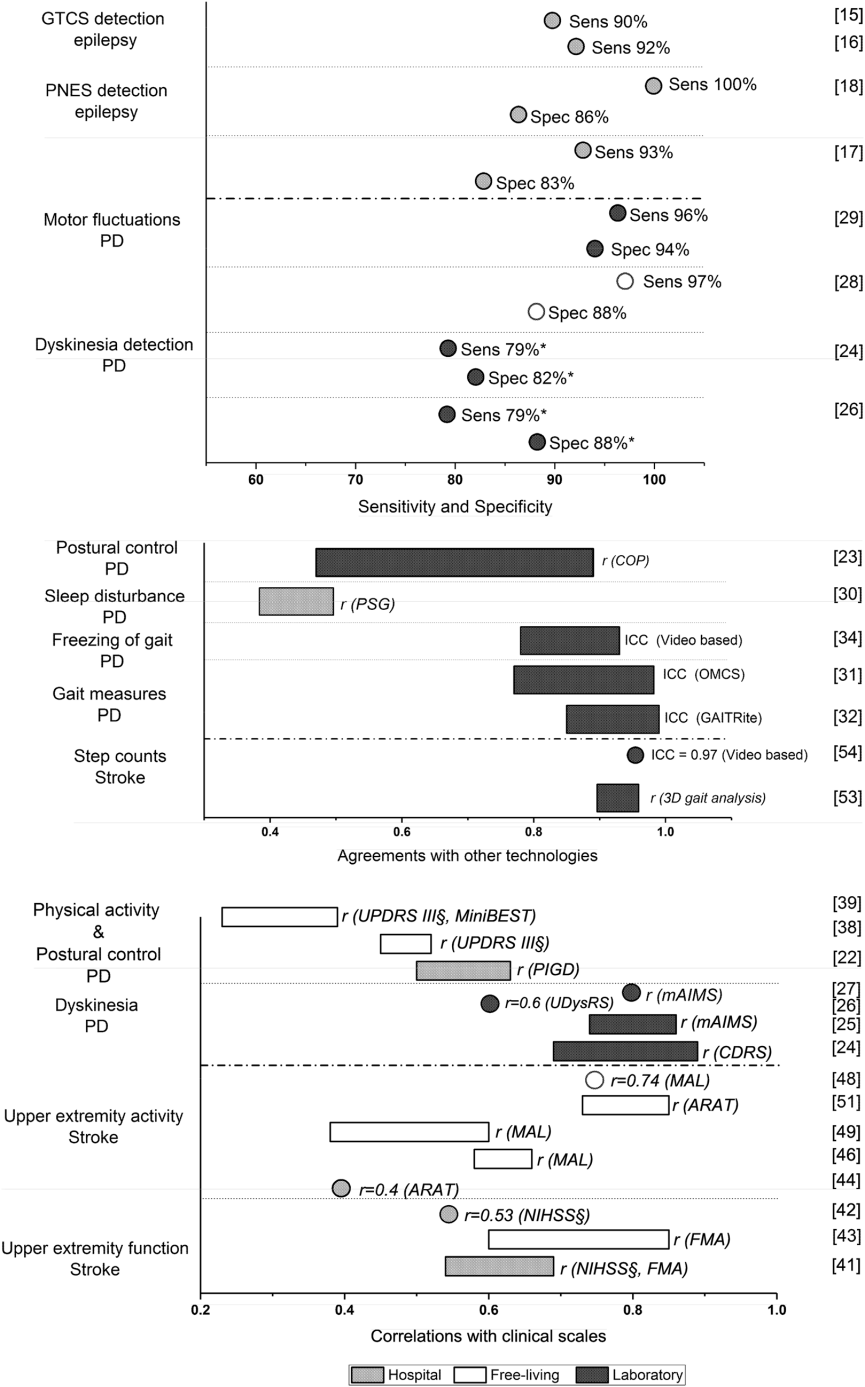
Reported outcomes of measures derived from wearables applied in epilepsy, PD, and stroke. *GTCS* generalized tonic–clonic seizures, *PNES* pshychogenic non-epileptic seizures, *PD* Parkinson’s disease, *Sens* sensitivity, *Spec* specificity, *COP* center of pressure, *ICC* intraclass correlations, *PSG* polysomnography, *OMCS* optical motion capture system, *ARAT* the Action Research Arm Test, *MAL* The Motor Activity Log, *FMA* Fugl–Meyer Assessment, *NIHSS* the Nation Institutes of Health Stroke Scale, *UPDRS* Unified Parkinson’s Disease Rating Scale, *MiniBEST* Mini Balance Evaluation Systems Test, *PIGD* postural instability and gait disorder, *UDysRS* Unified Dyskinesia Rating Scale, *mAIMS* modified Abnormal Involuntary Movement Scale, *CDRS* Clinical Dyskinesia Rating Scale. *Mean value is presented; §Negative correlation is shown

In laboratory settings, different standardized daily activities and functional walking and mobility tasks with more or less constrained protocols were used in studies with PD and stroke. Video observations, clinical scales, and other technologies such as gait analysis were often used as standard reference to validate variables derived from wearables. In PD, both accelerometers and gyroscopes were used, while step counts from accelerometers and energy expenditure during walking were investigated in stroke (Fig. 2 and Table 1).

**Table 1:** Clinimetric properties of measures derived from wearables in laboratory

Laboratory	Parkinson’s disease					Stroke
Validity	Medication-evoked adverse symptoms	Tremor	Gait measures	Freezing of gait	Postural control	Step counts
Discrimination, healthy/controls	[24, 26]	[20]	[31]		[22, 23]	[59] [54]
Discrimination, disease severity	[24, 26]					
*Standard references*						
Video-based ratings	[25–27]^a^, [29]^b^	[21]^a^		[33, 34]^c^		[54]^c^
Clinical assessment	[24]^a^				[23]^a^	
Visual observations						[59]^c^
Other technologies (gait analysis, center of pressure)			[31]^b^, [32]^a^		[23]^a^	[53]^c^
Reliability		[21]	[32]		[23]	[59]
Responsiveness		[21]			[22]	

^a^3-axial accelerometer and 3-axial gyroscope or inertial measurement units

^b^3-axial accelerometer

^c^1- or 2-axial accelerometer

Different measures derived from wearables quantifying tremor, dyskinesia, postural sway, and spatiotemporal gait characteristics discriminated well between individuals with PD and healthy controls [20, 22, 24, 26, 31]; dyskinesia measures discriminated also between patients with and without dyskinesia [24, 26]. Moderate-to-strong correlations were reported between dyskinesia detected from wearables and clinical ratings [24–26]. In addition, good agreement was found between sway and spatiotemporal gait measures from wearables and other established technologies [23, 31, 32]. Wearables showed good agreement with video-based ratings regarding the number of freezing episodes and the percentage of time with freezing of gait [34]. Postural sway measures derived from wearables have been examined for test–retest reliability (ICC 0.55–0.86) [23] and the mediolateral sway and jerk were shown to be sensitive to detect progression of postural instability in PD over time [22].

In stroke, good agreement was found between step counts derived from wearables compared to step counts from 3D gait analysis [53] or video-based counts [54]. One study reported no significant correlation between step counts derived from arm worn sensors and manual observational step counting, while an inconsistent but moderate-to-strong correlation (*r* = 0.56–0.85) for measuring energy expenditure was noted with indirect calorimetry [59]. Test–retest reliability for step counts and energy expenditure (ICC = 0.61–0.98) was also reported [59].

### Wearables in hospital environment

In hospital environments, patients were free to move and perform their daily activities within the ward or hospital. Only accelerometer data were reported and measurements lasted between 1 and 9 days. No studies investigated the test–retest reliability or responsiveness in free activities at hospital settings. Video electroencephalography (video-EEG), clinical scales, and polysomnography were used as the standard references to validate the variables derived from wearables (Fig. 2 and Table 2).

**Table 2:** Clinimetric properties of measures derived from wearables in free activities at hospital

Free activity in hospital	Epilepsy			Parkinson’s disease	Stroke	
Validity	Generalized tonic–clonic seizures	Psychogenic non-epileptic seizures	Motor seizures	Sleep disturbance	Upper extremity activity	Walking
Discrimination, healthy/controls					[41, 42, 44, 50]	
Discrimination, disease severity		[17, 18]			[40, 41]	
*Standard references*						
Video electroencephalogram	[15]^b^, [16]^c^	[17, 18]^b^	[14]^b^			
Clinical assessment					[40–42, 44]^c^	[61]^c^
Polysomnography				[30]^c^		
Reliability						
Responsiveness						

^a^3-axial accelerometer and 3-axial gyroscope or inertial measurement units

^b^3-axial accelerometer

^c^1 or 2-axial accelerometer

In epilepsy, stereotypical movement patterns for motor seizures were detected with three-axes accelerometers in 95% of the motor seizures identified with video-EEG [14]. More recent studies demonstrated detection sensitivity ranging from 90 to 92% for convulsive seizures [15, 16], but the false positive events varied between the studies. One study reported 40 false alarms in 16 out of 73 patients [15], and another study found 81 false alarms reported in 17 patients out of a sample of 30 [16]. Differentiation of psychogenic non-epileptic seizures from epileptic seizures showed a sensitivity of 93–100% with different machine learning approaches, while the specificity ranged from 75 to 91% [17, 18].

Upper extremity activity measures derived from accelerometers discriminated well between persons with stroke and healthy controls [41, 42, 44, 50] as well as between patients with different impairment levels [40, 41]. Moderate correlations were found between arm activity measures (activity counts) and clinical assessments in individuals with acute stroke [40–42, 44]. In one study, the walking activity measured with ankle accelerometers in hospital showed low correlation with stroke severity, but interestingly, a greater level of asymmetry was detected for individuals with stroke during their daily walking at hospital compared to laboratory gait analysis [61].

### Wearables in a free-living environment

Monitoring of movement related symptoms and deficits in a free-living environment is challenging. Differentiating or quantifying disease-related movement patterns like epileptic seizures from common voluntary movements such as teeth brushing can be challenging. To overcome these problems, advanced algorithm development is often required to reach sufficient accuracy. The wearing time in studies conducted in the free-living environment varied between 8 h and 7 days and only data from accelerometers were used. Clinical scales were commonly used to determine relationships between wearables and clinical assessments (Fig. 2 and Table 3).

**Table 3:** Clinimetric properties of measures derived from wearables in free-living environment

Free living	Parkinson’s disease				Stroke			
Validity	Bradykinesia	Medication-evoked adverse symptoms	Fall	Physical activity	Upper extremity activity	Physical activity and sedentary time	Step counts	Walking
Discrimination, healthy/controls	[19]	[28]			[45]	[56]		[60]
Discrimination, disease severity	[19]	[28]	[35, 36]	[38]	[43, 45, 47, 48]			[60]
*Standard references*								
Clinical assessment	[19]^c^			[38]^c^, [39]^b^	[43, 48, 49]^c^, [46, 51]^b^	[62]^c^		
Electrogoniometry					[45]^c^			
Laboratory tests						[57]^b^		
Reliability					[48, 49]	[56–58]	[52]	
Responsiveness				[37]		[55] [62],		[60]

^a^3-axial accelerometer and 3-axial gyroscope or inertial measurement units

^b^3-axial accelerometer

^c^1- or 2-axial accelerometer

In PD, acceleration-based assessment of bradykinesia in free-living settings was already described in 1998 [19]. The results showed that acceleration of extremities and immobility measures was effective to discriminate individuals with PD from controls [19]. A more recent study showed that a commercial proprietary algorithm could discriminate between individuals with and without motor fluctuations, and detect changes in fluctuations before and after deep brain stimulation [28]. Quantification of missteps and risk of falling was shown to discriminate non-fallers and fallers [35, 36]. A poor-to-moderate correlation was reported between measures from accelerometers (e.g., step counts and activity counts) and unified Parkinson’s disease rating scale [38, 39]. Over a 1-year period, a decline in physical activity levels was detected using accelerometers in individuals with PD [37].

In stroke, arm activity measures discriminated effectively between individuals with stroke and healthy controls [45], and between different motor impairment levels [45, 47, 48]. Moderate-to-strong correlations were found between accelerometer measures (threshold-based counts per time unit) and clinical upper extremity scales in chronic stroke [46, 49]. The test–retest reliability varied in different studies, but moderate agreements (ICC 0.54 and 0.68) were found for 3- and 7-day monitoring of daily activity counts [56]. Measures based on gait (e.g., step counts and step rate) over 1- or 3-day periods showed good test–retest reliability (ICC = 0.83–0.99) [52]. Threshold-based activity counts of arm activity were also shown to be reliable (r = 0.81–0.9) in test–retest [49]. Measures of activity levels (e.g., amount of time spent in an upright position) showed changes over time both during the acute and subacute stage of stroke [55]. The amount of time spend walking, standing, and number of walking bouts were also shown to be sensitive to change over a 12-week period after stroke [60].

### Adherence to wearables

**Fig. 3:**
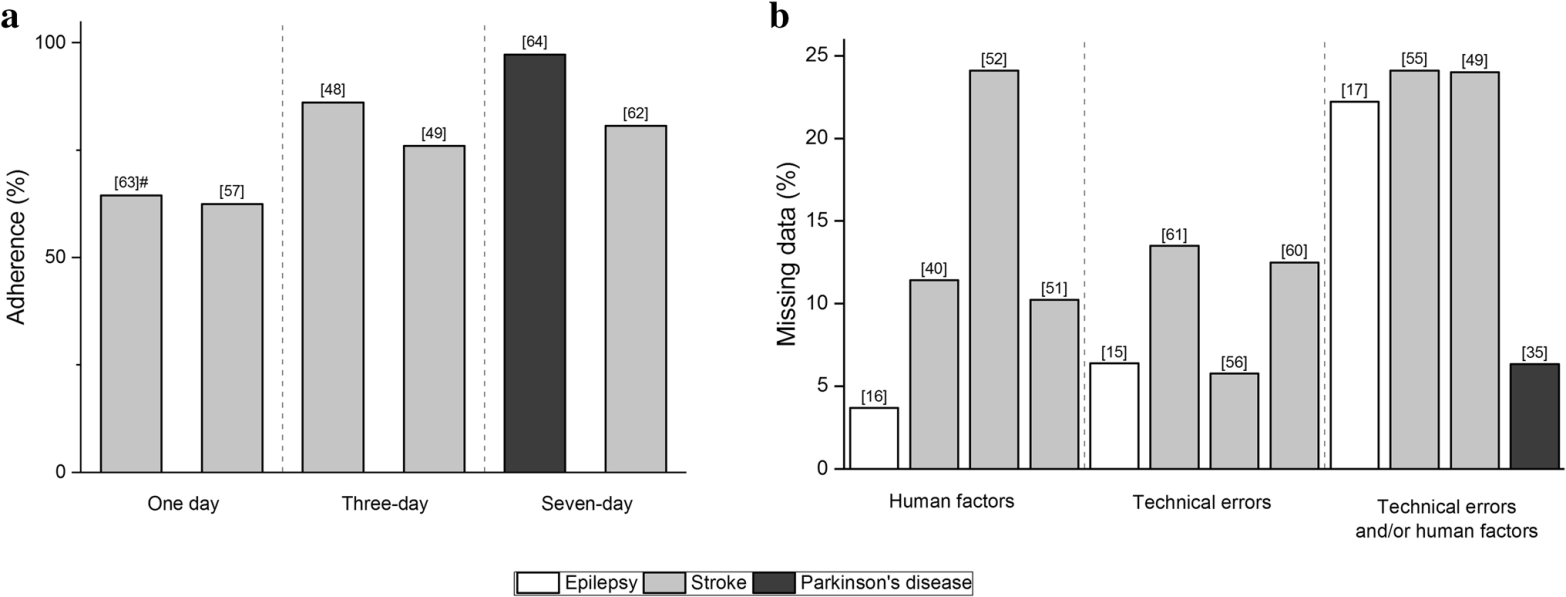
**a** Adherence of continuous monitoring using wearables. **b** Reported missing data due to technical errors and/or insufficient time of wearing or person related reasons. Mean data is presented. ^#^Adherence rate is shown

Five studies in stroke and one in PD have reported compliance regarding the use of wearables (Fig. 3a). A large study (*n* = 408) that investigating adherence to the use of step activity monitor over 2-day reported adherence rates between 61 and 68% for separate days, but only 53% of participants wore the sensors for two consecutive days [63]. Older individuals and those with better balance self-efficacy and walking endurance showed better adherence [63]. An intervention study with stroke showed that participants wore accelerometers 76–89% of waking hours in a 3-day measurement [48, 49]. A study evaluating acceptability of wrist-worn sensors in PD reported that only two persons of 34 did not wear the sensors for the full 7-day period and the non-adherence time was 4% [64].

### Missing and incomplete data

Missing data, as reported in 12 studies, was attributed to technical errors and/or human factors. Four studies reported technical errors including device failures, disconnection between sensors, and data storage problems (Fig. 3b). The average percentage of missing data attributable to technical errors in the reported studies was 10% (range 6–14%). Four studies reported that human factors, such as the device being removed and/or used incorrectly, were the predominant reasons for incomplete data. Missing data attributable to human factors were on average 12% (range 4–24%). The average of missing data resulted from both human factors and technical errors was 19% (range 6–24%).

### Studies reporting qualitative data

Three analytic themes emerged in the qualitative thematic synthesis: acceptable integration in daily life, lack of confidence in technology, and the need to consider individualization (Table 4).

**Table 4:** Thematic synthesis of patients’ experiences, acceptance, and preferences for use of wearables

Analytic themes	Descriptive themes	Subthemes
Acceptable integration in daily life	Acceptable properties	Acceptable different designs
		Acceptable long-term use
	Acceptable functions in daily life	Easy to don-off
		Comfortable in daily activities
Lack of confidence in technology	Psychosocial influence	Self-conscious in public
		Anxious in wearing technology
	Need for confirmation	Need for technical support
		Need for extra training
		Need for feedback
	Difficulties in use	Difficulties with correct use
		Difficulties to deal with technical failure
		Difficulties to manage battery
The need to consider individualization	User friendliness	Less obtrusive in appearance
		Easy to learn and use
	User benefits	Improvement for disease management

### Acceptable integration in daily life

In general, individuals with epilepsy, PD, and stroke were positive towards using wearables, such as body-worn small separate sensor units [64], gloves [65], smart glasses [66], and “intelligent” clothes [67]. Acceptable wearing time was reported to be 7 days for patients with PD [64]. Persons with epilepsy reported that they would agree to use a seizure registration device, and 65% would want to use it permanently [67]. Participants with stroke and PD described that wearables did not impact their daily activities [64, 65, 68, 69]. The participants found that wrist-worn sensors were easy to put on and take off [64]; however, in other studies, some participants with stroke felt that extra help would be needed to put the wrist sensors on but the sensors were comfortable to wear during daily activities [65, 68, 69].

### Lack of confidence in technology

Participants with PD and stroke were mostly positive and agreed to use wearables both at home and in public environments. Some felt self-consciousness using when they could be seen by others, especially during summer [64, 69]. A potential cause of embarrassment and stigmatization was anticipated when other people might ask or question what they were wearing, and in this way make their disease more apparent. Feeling “embarrassed” and that the sensors might “look funny” were described by participants with stroke [65]. Some participants also expressed feelings of stress and awkwardness towards the very idea of wearing a technological device [69]. PD participants further expressed that it was stressful to fasten the sensors during an off state [64, 69].

Participants worried that the sensors would get wet while washing dishes or showering [64, 69]. Participants with stroke felt a need for clear instructions on how to use the device, including both how to wear and how to operate it [68]. They wanted repeated instructions, confirmation, supervised practice, and external support for technical problems in follow-up sessions to improve their confidence [68, 69].

In addition, participants with PD and stroke reported difficulties in using the device correctly, handling technical errors, and charging the battery. They worried that unpredictable technical errors would lead to confusion about how to handle the wearables [68]. They experienced that keeping and placing the sensors at correct positions were difficult [64, 68, 69], and in PD, this was even more challenging during an off state [64, 69].

### The need to consider individualization

Individuals with epilepsy, PD, and stroke reported a wide spectrum of expectations in terms of usability of wearables [65–69]. Participants with PD and stroke described that wearables should be easy to learn and use [65, 66, 68, 69]. Wearables need to be small and non-obtrusive [64, 65, 68, 69], and some stroke participants suggested that sensors could be worn on the upper arm instead of the wrists to make them less noticeable [65]. Both epilepsy and PD participants further described desirable features of wearables, including the possibility of real-time analysis of data, getting reminders to take drugs and waterproof design [66, 67, 69]. Persons with epilepsy wanted features that would allow improved diagnosis and seizure management [67]. PD participants wanted wearables to assist with physiotherapy training, to improve gait and balance problems [66].

## Discussion

This systematic review illustrates how wearables have been used to monitor movement and disease-related signs in epilepsy, PD, and stroke in different environments, including laboratory, hospital, and free-living. Despite an increasing number of studies using wearables in clinical applications, only half of the eligible studies identified were of sufficient reporting quality. In epilepsy, the wearables were primarily used to detect and differentiate seizures. In PD, the focus was on quantification of dyskinesia, tremor, and bradykinesia, and in stroke, the focus was on upper extremity activity, gait, and physical activity. Clinimetric properties were predominantly investigated in studies using discrete outcome variables such as activity counts or other acceleration-derived variables, in contrast to studies, where complicated algorithms were developed and in which the correct classification and precision of these algorithms were usually tested. The validity of measures derived from wearables was to some extent addressed in several studies, but the reliability and responsiveness have only been studied in PD and stroke. For example, the postural sway measures in PD have been shown to be reliable and sensitive to longitudinal changes in laboratory settings [22, 23]. In stroke, the step counts and measures of upper extremity and physical activity were shown to be reliable [48, 49, 52, 56–59], and sedentary or upright and walking behaviour measures have been shown to be sensitive to longitudinal changes [55, 60, 62].

The current review also showed that technical errors and human factors influenced adherence and are important reasons for loss of data. The qualitative thematic analysis of studies which reported users’ experiences and acceptance rendered three main analytic themes: acceptable integration in daily life, lack of confidence in technology, and the need to consider individualization. These themes reflect some challenges that need to be met for wearables to be integrated in the clinical practice.

This review included 22 studies conducted in free-living environments, 16 studies in laboratory, and 12 in hospital settings. Data collection in a standardized environment such as laboratory and hospital allows a more detailed evaluation of algorithm and device performance during well-defined movements and tasks in comparison with other established methods like video, optical motion capture or EEG. The evaluation is much more challenging in complex and unpredictable free-living conditions. As a reflection of this, we found no studies on epilepsy based on measurements during free-living conditions. In some cases, it may be possible to “move the laboratory” into free-living environment as a transition strategy to confirm device and algorithm performance as free-living conditions carries the most promising potential of wearables, e.g., with wearable EEG equipment and video monitoring in predefined areas. In the long run, however, evaluation of the performance of wearables in free-living conditions will have to include interventional studies that address the effect of using automatic home-monitoring on disease-related endpoints. At some stage, the transition from laboratory to clinical use will, therefore, involve a leap of faith, where one has to be convinced that the devices and algorithms are good enough to be used in randomized clinical trials. The lack of data on clinical utility of using wearables in free-living conditions is a gap that needs to be filled. Promising results have been reported for capturing motor fluctuations in PD using a single wrist sensor [28] and correctly classifying individuals with dyskinesia using a single ankle-worn sensor in home environment [26]. As these phenomena influence quality of life and can be influenced by changes in treatment, improved detection and evaluation with wearables can be expected to improve disease-specific quality of life and other measurements of disease burden.

Several studies in PD and stroke using wearables during free activities or in free-living conditions reported moderate-to-strong correlations between measures derived from wearables and clinical scales. Although the clinical scales may adequately reflect the patients’ symptoms or disabilities, they are often limited by the predefined ordinal scoring levels and lack sensitivity to more detailed and subtle changes in the clinical status [22, 37, 70, 71]. For the detection of seizures, which are relatively rare and brief events, the requirement for accuracy is greater than for detecting symptoms of PD and activity measures in stroke. It is, therefore, a bigger step to move from controlled to uncontrolled environments. One thing common to the three disorders, however, is that during free-living monitoring, the comparison methods are often subjective and retrospective. The challenge of evaluating wearable devices without a reasonably good reference needs to be addressed before they can be applied in regular care.

Predominantly acceleration signals were used in hospital and free-living settings, even though gyroscope signals have shown promise for increasing the sensitivity and specificity when measuring dyskinesia and postural instability in laboratory settings [23–26]. One explanation is that gyroscope signals consume more battery power and in this way limit the measuring time. One epilepsy study has suggested that the use of electrodermal activity together with accelerometry might increase sensitivity and specificity in seizure detection, compared to the use of accelerometry only [72]. The idea of measuring multiple physiological modalities can also be transferred to the detection of non-motor symptoms in PD. The practical problems of processing and storing large volumes of data will, however, increase with the use of multiple sensors and modalities. To improve precision, patient-specific algorithms have recently been suggested in epilepsy [73].

Interestingly, we found that in studies where the monitoring time was longer, better adherence to wearables was reported. This could indicate that increased confidence with the use of wearables could have a positive impact on adherence. The lack of confidence in handling the new technology was also one of the main themes that emerged from our thematic synthesis. Optimal wearing time will also vary depending on the nature of the symptoms targeted. For example, 1 month or more could be needed for monitoring seizures in an epilepsy outpatient. For monitoring motor fluctuations in PD or physical activity levels in stroke, 7 days would be ideal because of expected variations between activity during weekdays and weekends, although 1–3 days may be more practical.

Human factors contributed to between 4 and 24% of data loss in the included studies. For routine use, data loss has to be in the lower part of this range and it is, therefore, important to analyse which factors are most important for non-adherence. A positive acceptance towards the use of wearables emerged as one of the main themes from our thematic synthesis and technical support and feedback were considered important factors for increasing motivation and confidence in the use of wearables.

This systematic review, like several before [70, 74–78], highlights a need to further investigate the clinimetric properties of the measures derived from wearables, to improve standardization of data protocols, variable definitions, and to encourage further development of patient-specific algorithms. The possible benefit of using multimodal information needs to be further investigated. After validating devices and algorithms in controlled environment efforts should be made to subject the wearable technology to randomized clinical trials that can determine if home-monitoring improves management and treatment results. This review also reveals a need to improve the reporting quality of studies evaluating wearables for clinical applications, which would improve dissemination of results into clinical practice. We identified a wide range of outcome measures, but no studies directly addressed the question of the effect wearables may have on decision making or clinical treatment outcomes. The clinical utility, therefore, remains to be established.

## Electronic supplementary material

Below is the link to the electronic supplementary material.

Supplementary material 1 (PDF 256 kb)

Supplementary material 2 (XLSX 35 kb)

Supplementary material 3 (DOCX 41 kb)
