# Supplementary material for: Wearable sensors for clinical applications in epilepsy, Parkinson’s disease, and stroke: a mixed-methods systematic review
Source: J Neurol. 2018 Feb 9;265(8):1740–52. doi: 10.1007/s00415-018-8786-y (PMC6060770; doi:10.1007/s00415-018-8786-y)
Supplement: Supplementary file 1 — Supplementary material 1 (PDF 256 kb) [file 415_2018_8786_MOESM1_ESM.pdf]

Wearable sensors for clinical applications in epilepsy, Parkinson's disease and stroke: A mixed-methods systematic review

Journal of Neurology

Dongni Johansson[1], Kristina Malmgren[1], Margit Alt Murphy[1]
[1]Dept. of Clinical Neuroscience, Institute of Neuroscience and Physiology, Sahlgrenska Academy, University of Gothenburg, Gothenburg, Sweden

Correspondence: Dongni Johansson, Department of Clinical Neuroscience, Institute of Neuroscience and Physiology, Sahlgrenska Academy, University of Gothenburg, Gothenburg, Sweden

Quantitative studies searched in PubMed

Population	Search Strategy: (Epilepsy, Tonic-Clonic/diagnosis* [MeSH Terms] OR seizures/diagnosis*[MeSH Terms] OR Parkinson Disease/diagnosis*[MeSH Terms] OR Parkinson Disease/complication*[MeSH Terms] OR Stroke/rehabilitation*[MeSH Terms] OR Movement Disorders/diagnosis*[MeSH Terms]OR ”Parkinson’s disease” OR ”Parkinson disease” OR epilepsy OR seizure* OR stroke OR “movement disorders” NOT Stroke Volume/physiology[MeSH Terms] NOT Stroke Volume*[MeSH Terms] NOT child[MeSH Terms])
Intervention	Search Strategy: (“wearable sensor*”[tiab] OR accelerometer[tiab] OR accelerometry[tiab] OR Monitoring, Physiologic/instrumentation*[MeSH Terms] OR Accelerometry/methods*[MeSH Terms] OR Wireless Technology*[MeSH Terms] OR “on-body sensors” [tiab] OR inertial[tw] OR gyroscope[tw] NOT smartphone NOT mobile NOT Electrodes,Implanted*[MeSH Terms] NOT Prostheses and Implants[MeSH Terms] NOT Doppler[tw] NOT Monitoring,Intraoperative/instrumentation*[MeSH Terms])
Comparison	Search Strategy: (N/A)
Outcome	Search Strategy: (Motor activity*/physiology[MeSH Terms] OR Motion [MeSH Terms] OR Monitoring, Physiologic/methods*[MeSH Terms] OR Movement/physiology*[MeSH Terms] OR vital sign*/physiology[MeSH Terms] OR bradykinesia[tiab] OR dyskinesia[tiab] OR fluctuation[tiab] OR ”levodopa dose”[tiab] OR UPDRS[tw] OR “Sensitivity and Specificity”[MeSH Terms] OR sensitivity[tw] OR specificity[tw] OR “Activities of daily living”[tw] OR FMA[tw] OR gait[tw] OR tremor[tw] OR “upper limb ”[tw] OR “lower extremity”[tw] OR function[tw] OR accuracy[tw] OR “seizure detection” [tw] OR postural stability[tw] OR AIMS[tw] OR algorithm[tw] OR “time frequency” [tw] NOT “left ventricular”[tw] NOT intraoperative[tiab] NOT Essential Tremor/drug therapy*[MeSH Terms] NOT Tremor/drug therapy*[MeSH Terms])) AND (patient*[tw] OR person OR participant*[tw] OR individuals[tw] OR subjects[tw]) AND (validation[tw] OR validity[tw] OR reliability[tw] OR evaluation[tw] OR responsiveness[tw] OR comparability[tw] OR effectiveness[tw] OR reproducibility[tw] OR “Sensitivity and Specificity”[MeSH Terms] OR sensitivity[tw] OR specificity[tw]))
Search strategy	(((Epilepsy, Tonic-Clonic/diagnosis* [MeSH Terms] OR seizures/diagnosis*[MeSH Terms] OR Parkinson Disease/diagnosis*[MeSH Terms] OR Parkinson Disease/complication*[MeSH Terms] OR Stroke/rehabilitation*[MeSH Terms] OR Movement Disorders/diagnosis*[MeSH Terms]OR ”Parkinson’s disease” OR ”Parkinson disease” OR epilepsy OR seizure* OR stroke OR “movement disorders” NOT Stroke Volume/physiology[MeSH Terms] NOT Stroke Volume*[MeSH Terms] NOT child[MeSH Terms]) AND (“wearable sensor*”[tiab] OR accelerometer[tiab] OR accelerometry[tiab] OR Monitoring, Physiologic/instrumentation*[MeSH Terms] OR Accelerometry/methods*[MeSH Terms] OR Wireless Technology*[MeSH Terms] OR “on-body sensors” [tiab] OR inertial[tw] OR gyroscope[tw] NOT smartphone NOT mobile NOT Electrodes,Implanted*[MeSH Terms] NOT Prostheses and Implants[MeSH Terms] NOT Doppler[tw] NOT Monitoring,Intraoperative/instrumentation*[MeSH Terms]) AND (Motor activity*/physiology[MeSH Terms] OR Motion [MeSH Terms] OR Monitoring, Physiologic/methods*[MeSH Terms] OR Movement/physiology*[MeSH Terms] OR vital sign*/physiology[MeSH Terms] OR bradykinesia[tiab] OR dyskinesia[tiab] OR fluctuation[tiab] OR ”levodopa dose”[tiab] OR UPDRS[tw] OR “Sensitivity and Specificity”[MeSH Terms] OR sensitivity[tw] OR specificity[tw] OR “Activities of daily living”[tw] OR FMA[tw] OR gait[tw] OR tremor[tw] OR “upper limb ”[tw] OR “lower extremity”[tw] OR function[tw] OR accuracy[tw] OR “seizure detection” [tw] OR postural stability[tw] OR AIMS[tw] OR algorithm[tw] OR “time frequency” [tw] NOT “left ventricular”[tw] NOT intraoperative[tiab] NOT Essential Tremor/drug therapy*[MeSH Terms] NOT Tremor/drug therapy*[MeSH Terms])) AND (patient*[tw] OR person OR participant*[tw] OR individuals[tw] OR subjects[tw]) AND (validation[tw] OR validity[tw] OR reliability[tw] OR evaluation[tw] OR responsiveness[tw] OR comparability[tw] OR effectiveness[tw] OR reproducibility[tw] OR “Sensitivity and Specificity”[MeSH Terms] OR sensitivity[tw] OR specificity[tw]))

Qualitative studies searched in Scopus

**Population**	Search Strategy: epilepsy OR parkinson OR "Parkinson Disease" OR stroke OR seizure*
**Intervention**	Search Strategy: TITLE-ABS-KEY (accelerometer) OR TITLE-ABS-KEY ("wearable sensors" OR "wireless sensors" OR "body worn sensors" AND wearable OR smart AND cloth) OR TITLE-ABS-KEY ("wearable sensors" OR "wireless sensors" OR "body sensors") AND NOT mobile
**Comparison**	N/A
**Outcome**	Search Strategy: ("focus group interview" OR "focus group discussion" OR interviews OR survey OR questionnaires OR qualitative OR "prefe rence" OR compliance OR acceptance OR needs OR satisfaction OR barriers OR f acilitator OR "User-centered design")
**Search strategy**	((epilepsy OR parkinson OR "Parkinson Disease" OR stroke OR seizure*) AND (TITLE-ABS-KEY (accelerometer) OR TITLE-ABS-KEY ("wearable sensors" OR "wireless sensors" OR "body worn sensors" AND wearable OR smart AND cloth) OR TITLE-ABS-KEY ("wearable sensors" OR "wireless sensors" OR "body sensors") AND NOT mobile) AND ("focus group interview" OR "focus group discussion" OR interviews OR survey OR questionnaires OR qualitative OR "prefe rence" OR compliance OR acceptance OR needs OR satisfaction OR barriers OR f acilitator OR "User-centered design")) AND (EXCLUDE (PUBYEAR , 2018) OR EXCLUDE (PUBYEAR , 2 017) OR EXCLUDE (PUBYEAR , 1994) OR EXCLUDE (PUBYEAR , 1993) O R EXCLUDE (PUBYEAR , 1991) OR EXCLUDE (PUBYEAR , 1988) OR EXCL UDE (PUBYEAR , 1987) OR EXCLUDE (PUBYEAR , 1986) OR EXCLUDE (P UBYEAR , 1983) OR EXCLUDE (PUBYEAR , 1982) OR EXCLUDE (PUBYEA R , 1981) OR EXCLUDE (PUBYEAR , 1979) OR EXCLUDE (PUBYEAR , 1976)) AND (EXCLUDE (SUBJAREA , "ENGI") OR EXCLUDE (SUBJAREA , "CO MP") OR EXCLUDE (SUBJAREA , "BIOC") OR EXCLUDE (SUBJAREA , "AG RI") OR EXCLUDE (SUBJAREA , "PHYS") OR EXCLUDE (SUBJAREA , "MA TE") OR EXCLUDE (SUBJAREA , "MATH") OR EXCLUDE (SUBJAREA , "C HEM") OR EXCLUDE (SUBJAREA , "CENG") OR EXCLUDE (SUBJAREA , " ENVI") OR EXCLUDE (SUBJAREA , "ARTS") OR EXCLUDE (SUBJAREA , " EART") OR EXCLUDE (SUBJAREA , "IMMU") OR EXCLUDE (SUBJAREA , " PHAR") OR EXCLUDE (SUBJAREA , "DECI") OR EXCLUDE (SUBJAREA , " BUSI") OR EXCLUDE (SUBJAREA , "ENER") OR EXCLUDE (SUBJAREA , " ECON") OR EXCLUDE (SUBJAREA , "VETE")) AND (LIMIT-TO (LANGUAGE , "English")) AND (LIMIT-TO (DOCTYPE , "ar")) AND (LIMIT-TO (SRCTYPE , "j"))

Qualitative studies searched using SPIDER tool in Scopus

**Sample**	Search Strategy: MeSH Terms: (Epilepsy, Tonic-Clonic/diagnosis* OR seizures/diagnosis* OR Parkinson Disease/diagnosis* OR Parkinson Disease/complication* OR Stroke/rehabilitation* OR Movement Disorders/diagnosis*)
**Phenomenon of Interest**	Search Strategy: (wearable sensors[tiab] OR accelerometer[tiab] OR Monitoring, Physiologic/instrumentation*[MeSH Terms] OR Accelerometry/methods*[MeSH Terms] OR Wireless Technology* [MeSH Terms])
**Design**	Search Strategy: ("focus groups" OR interviews OR survey OR Questionnaires[MeSH Terms] OR Data Collection[MeSH Terms])
**Evaluation**	Search Strategy: (Patient Preference*[MeSH Terms] OR Perception*[MeSH Terms] OR Equipment Design*[MeSH Terms] OR User-centered design OR User preferences[tiab] OR acceptance[tw] OR needs[tw] OR satisfaction[tw] OR barriers[tw])
**Research Type**	Search Strategy:(qualitative[tw] OR Qualitative Research[MeSH Terms])

Search Strategy: (((Epilepsy, Tonic-Clonic/diagnosis* [MeSH Terms] OR seizures/diagnosis*[MeSH Terms] OR Parkinson Disease/diagnosis*[MeSH Terms] OR Parkinson Disease/complication*[MeSH Terms] OR Stroke/rehabilitation*[MeSH Terms] OR Movement Disorders/diagnosis*[MeSH Terms] OR Stroke OR epilepsy OR seizures OR Parkinson OR "Movement disorders") AND (wearable sensors[tiab] OR wireless sensors[tiab] OR body worn sensors[tiab] OR accelerometer[tiab] OR Monitoring, Physiologic/instrumentation*[MeSH Terms] OR Accelerometry/methods*[MeSH Terms] OR Wireless Technology* [MeSH Terms]) AND (Patient Preference*[MeSH Terms] OR Perception*[MeSH Terms] OR Equipment Design*[MeSH Terms] OR "User-centered design" OR "User preferences"[tiab] OR acceptance[tw] OR needs[tw] OR satisfaction[tw] OR barriers[tw]) AND ("focus groups" OR interviews[tiab] OR survey OR Questionnaires[MeSH Terms] OR Data Collection[MeSH Terms]) AND (qualitative[tw] OR Qualitative Research[MeSH Terms]))
