# Supplementary material for: Wearable sensors for clinical applications in epilepsy, Parkinson’s disease, and stroke: a mixed-methods systematic review
Source: J Neurol. 2018 Feb 9;265(8):1740–52. doi: 10.1007/s00415-018-8786-y (PMC6060770; doi:10.1007/s00415-018-8786-y)
Supplement: Supplementary file 3 — Supplementary material 3 (DOCX 41 kb) [file 415_2018_8786_MOESM3_ESM.docx]

# Wearable sensors for clinical applications in epilepsy, Parkinson’s disease and stroke: A mixed-methods systematic review

# Journal of Neurology

Dongni Johansson^1^, Kristina Malmgren^1^, Margit Alt Murphy^1^

^1^Dept. of Clinical Neuroscience, Institute of Neuroscience and Physiology, Sahlgrenska Academy, University of Gothenburg, Gothenburg, Sweden

Correspondence: Dongni Johansson, Department of Clinical Neuroscience, Institute of Neuroscience and Physiology, Sahlgrenska Academy, University of Gothenburg, Gothenburg, Swed

**Supplementary Table 1** Overview of the studies included for data extraction sorted after the clinical applications area

| **Study** | **Sample size** | **Age**  **mean±SD**  **median(range)** | **Number of detected seizure events** | **Number of sensors/**  **placements** | **Methods and main variables** | | **Main findings** |
| --- | --- | --- | --- | --- | --- | --- | --- |
| ***Detection of motor seizures, epilepsy*** | | | | | | | |
| Nijsen 2005 [14] | 18 | 37±12 | 897 motor seizures | 5/ upper and lower limbs, trunk | Algorithm based; Video-EEG and observation | | Detection rate 428/897 (48%); Acceleration detected seizures only with motor phenomena and had no value for non-motor seizures; 95% of motor seizures showed stereotypical pattern. |
| ***Detection of GTCS, epilepsy*** | | | | | | | |
| Beniczky 2013 [15] | 73 | 36±14 | 39 GTCS  149 non-GTCS | 1/wrist | Commercial algorithm, video-EEG | | Detection rate 35/39 in 20 patients; Sens 0.9; False alarms 0.2/day; Detection latency 55s |
| Velez 2016 [16] | 27 | 41±14 | 13 GTCS  49 non-GTCS | 1/wrist | Commercial algorithm, video-EEG | | Detection rate (wristwatch) 12/13 in 10 patients; Sens 0.92; Audio recordings 11/12, Sens 0.92, False positives 81 |
| ***Classification of PNES and ES, epilepsy*** | | | | | | | |
| Bayly 2013 [17] | 99 | PNES 38 (19-83)  ES 33 (20-69) | 45 PNES  11 ES | 1/wrist | Machine learning (Time-frequency mapping- Fast Fourier transformations ), video-EEG | | Detection of PNES 38/41, Sens 0.93; ES 6/8, Spec 0.75, PPV 0.95,NPV 0.667; Coefficient of variation of time-frequency map detected 42/45 PNES, Sens 0.93; ES 10/11, Spec 0.91,PPV 0.98,NPV 0.77 in 35 patients |
| Gubbi 2015 [18] | 27 | 34±13 | 21 PNES  13 ES | 2/wrists | Machine learning (k-means clustering, time-frequency analysis, Support vector machines ) | | Sens 1.00, Spec 0.86 for 8 patients with 19 convulsive seizures, overall accuracy 0.92; Leave one out error of 6.67% for PNES classification |
| ***Bradykinesia, PD*** | | | **Disease duration, years,**  **mean±SD** | **Number of sensors/**  **placements** | **Methods and main variables** | | **Main findings** |
| Dunnewold 1998 [19] | Part I 15 ;  Part II 50,  43 C | Part I 58±14  Part II 59 ±12  C 63±13 | Part I 8±6;  Part II 6±5 | Part I 2/thigh, trunk  Part II 6/thighs, wrists, trunk | Algorithm based (discriminant analysis to determine the threshold of mean duration of immobility), UPDRS III | | Higher immobility, less time upright, more time lying, lower mean values of limbs acceleration in PD; No or low correlation with UPDRS III; Differences in gender on bradykinesia measures |
| ***Medication evoked adverse symptoms , PD*** | | | | | | | |
| Lopane 2015 [24] | 46  18 De novo  18 C | 63±9  De novo59±9  C 60±11 | 6±6  De novo 2±1 | 1/lower back | | Algorithm (linear discriminant analysis), CDRS | Dyskinesia recognition: Sens 0.69-0.88, Spec 0.68-0.97; Correlation with CDRS r=0.69-0.89; Test-retest ICC= 0.74-0.92 (controls and de novo PD) |
| Pulliam 2014 [25] | 15 | Range 42-72 | Range 3.5-17 | 6/ wrists, thighs, ankles | | Machine learning (iterative forward selection algorithm, 18 time- and frequency-domain measures), mAIMS | Acceptable accuracy of dyskinesia predictions with only two sensor on affected limbs; Correlation with mAIMS r=0.74-0.86 |
| Ramsperger 2016 [26] | 23 Lab,  13 C;  10 FreeAct | Lab: 68± 5  C 60±8  FreeAct 62±7 | 12±5 Lab:  12±4 FreeAct | Lab: 2/ankles  FreeAct: 1/most affected ankle | | Algorithm based (combined axis rotations), severity of LID, UDysRS | Lab: severity of LID: Sens 0.73-0.85,Spec 0.78-0.98; UDysRS r= 0.61; FreeAct: detection of LID 100% |
| Mera 2013 [27] | 15 | 59±11 | 10±5 | 2/wrists | | Kinematic features (power spectrum area and peak power within specified frequency band, KinetiSense motor sensor unit), mAIMS | The highest correlation r= 0.81with root mean square error 0.55 between the logarithm of the power spectrum area 0.3-3 Hz and the combined clinician mAIMS scores |
| Horne 2015 [28] | 527  38 C | 66±8  C (45-85) | 7 | 1/ most affected wrist | | Commercial algorithm (Parkinson’s KinetiGraph), fluctuation score reflecting variations in dyskinesia and bradykinesia scores. | Receiver operating characteristic: 0.98; Sens 0.971; Spec 0.875 |
| Rodriguez-Molinero 2015 [29] | 20 Lab,  15 FreeAct | Mean 64 (55-75) | Median 9.5 (IQR 2-18) | 1/waist | | Support vector machines algorithm with a linear kernel to detect ON/OFF; Lab: compared to video; FreeAct: compared to observations | Lab : Mean Sens 0.96; Mean Spec 0.94;  FreeAct (3 to 5 hrs): Sens 0.91; Spec 0.90; PPV 0.80; NPV 0.94 |
| ***Tremor, PD*** | | | | | | | |
| Scanlon 2013 [20] | 16  8 C | 64±9  C 56±9 | 10±11 | 2/ finger and leg | Commercial system (sensing stylus); Tremor intensity, center frequency, and intra-individual variability of center frequency | | Center frequency was lower in lower limbs than upper limbs. Less intra-individual variability of center frequency (rest and postural tremor in the dominant lower limb) in PD |
| Heldman 2014 [21] | 18 | 63±8 | Not reported | 1/ most affected index finger | Commercial system (Kinesia), UPDRS, MBRS | | Reliability of system: ICC 0.63-0.94 in finger tapping speed, amplitude and rhythm significantly higher than clinician ratings; Minimal detectable change was lower for Kinesia measures; No significant difference for detection of resting and postural tremor |
| ***Sleep disturbance, PD*** | | | | | | | |
| Maglione 2013 [30] | 61 | 67±9 | H&Y stage 2±1 | 1/non-dominant wrist | Actigraph (Actiwatch-L); Number of activity counts with epoch thresholds; Polysomnography | | Activity threshold of 10 was best; *Polysomnography*: r= 0.38-0.50. Higher H&Y stage showed greater difference in total sleep time, sleep efficiency, wake after sleep onset |
| ***Gait measures, PD*** | | | | | | | |
| Lord 2008 [31] | 12  11 C | 71±3  C 73±3 | 8±3 | 5/arms, legs, trunk | Vitaport Activity Monitor and GAITRite, gait speed, step length and frequency | | Agreement: ICC 0.85-0.99; Largest difference in step frequency |
| Esser 2012 [32] | 10 | 59±12 | 5±3 | 1/lower back (L4) | Projected center of mass, spatio-temporal gait variables during 10 meter walking | | Agreement and accuracy: No difference for positions, velocity and acceleration between systems; ICC=0.77-0.98 for step time, stride length and walking speed |
| ***Freezing of gait, PD*** | | | | | | | |
| Yungher 2014 [33] | 14 | 71±9 | 10±10 | 7/thighs, shanks, mid-feet, lower back | A time-frequency analysis to detect onset of leg oscillation | | A distal to proximal progression of oscillation prior FOG, oscillations were damped at the torso |
| Morris 2012 [34] | 10 | 68±7 | 11±7 | 2/ shanks | FOG (algorithm based) and clinical rating of FOG from video | | Agreement: number of FOG ICC: 0.78; Percentage time in FOG ICC: 0.93 |
| ***Fall, PD*** | | | | | | | |
| Weiss 2014 [35] | 107 | 65±9 | 6±3 | 1/ lower back | Algorithm based; Walking quantity and quality; | | Walking quality differed between fallers and non-fallers; No significant difference in walking quantity; Fallers have higher gait variability; Large variability in anterior-posterior width associated with higher risk of experiencing a fall |
| Iluz 2014 [36] | 40 | 62±10 | 5±4 | 1/ lower back | Algorithm based (gait detection; suspected misstep) | | Prior fall history showed increased likelihood of suspected missteps (Odds Ratio = 1.84) compared to a non-faller |
| ***Postural sway, PD*** | | | | | | | |
| Mancini 2012a,b [22,23] | Part I 13  12 C;  Part II 17 17 C | Part I PD 60±9, C 60±8;  Part II PD 67±7,C 68±6 | Part I: recently diagnosed, no medication at baseline; Part II: early, medication on | 1/lower back (L5) | Algorithm based 4 sway measures (root mean square, mean velocity, frequency, jerk) from Isway (MTX Xsens); | | Mediolateral sway measures and jerk were different from controls; Standardized response mean = 0.53-0.90 |
|  |  |  |  |  | Algorithm based 13 time/frequency measures; Isway compared with center of pressure (force plate) in 30s standing, UPDRS III, PIGD | | Jerk of the sway best to discriminate; Test-retest: jerk ICC 0.86; Time-domain measures ICC 0.55-0.84; PIGD, 8 of 13 Isway measures r=0.50-0.63; Center of pressure : r=0.47-0.89; No significant correlation with UPDRS III: r= 0.09-0.3; |
| ***Physical activity, PD*** | | | | | | | |
| Cavanaugh 2012 [37] | 33 | 67±9 | 4±4 | 1/ less- affected ankle | Commercial system (SAM) | | Sensitive to detect decline over time for step counts, peak activity index, maximum output, time of moderate intensity |
| Skidmore 2008 [38] | 26 | 70±9 | 8±4 | 1/ right ankle | Commercial system (SAM); UPDRS and H&Y stage | | Strongest correlation: number of steps/day, steps/min in the most active time; Correlation with UPDRS I r= -0.39; UPDRS III r= -0.45 to -0.52. H&Y stage 3 had lower activity levels and step rate |
| Nero 2016 [39] | 91 | 73±60 | 5 (IQR 2-8) | 1/lower back | Commercial system (ActiGraph); Correlation with demographic, disease and mobility-related factors, UPDRS III, gait measures from GAITRite, PDQ-39, MiniBESTest, SF-36 | | Correlation: total activity counts /day, minutes of walking (>1.0 m/s); UPDRS III: r= -0.23 -0.25; GAITRite: r= 0.34-0.41; MiniBESTest: r=0.32-0. 39; UPDRS III, SF-36 (physical function),BMI and dyskinesia explained 34% of total activity counts; SF-36 (physical function ) and MiniBESTest score explained 22% of brisk walking time |
| ***Physical activity and sedentary time, stroke*** | | | | | | | |
| Askim 2013 [55] | 28 | 79±9 | 8±3 days, 1,3,6 months | 2/ right thigh, calf | Commercial system (PAL); Responsiveness for time in lying, standing, transition over 4 test periods. | | Mean time in upright position increased from 92 to 144 min during 6 months recovery; Change in BBS and Barthel Index were associated with increase in upright position |
| Hale 2008 [56] | 20  9 C | 72±7 | Not reported | 1/waist | Commercial system (TriTrac RT3); activity counts (3 and 7 days) | | Test-retest reliability: 7-day ICC=0.68-0.85; 3-day ICC=0.54-0.97; The absolute reliability of 7-day data = 23%; Correlation with 7-day recall questionnaire: r=0.61 |
| Rand 2009 [57] | 40 | 67±10 | 3±6 | 2/ iliac spines | Commercial system (Actical), total activity counts/day and total energy expenditure/day. | | Activity counts: paretic ICC= 0.95, non-paretic ICC=0.94, between paretic and non-paretic ICC=0.98; Energy expenditure: paretic ICC=0.95, non-paretic ICC=0.95, between paretic and non-paretic ICC=0.96; Correlation with 6MWT: r= 0.6-0.73 |
| Haeuber 2004 [58] | 17 | 65±6 | 42 months | 1/ non-paretic ankle | Commercial system (SAM); strides/day over two 48 hrs periods; Energy expenditure (Caltrac) | | Test-retest reliability: strides /24hrs r= 0.96, energy expenditure (Caltrac) r=0.44 and not significant; Correlations between energy expenditure (Caltrac) and SAM derived strides r=0.77-0.82 |
| Vanroy 2014 [59] | 15  15 C | 60±10  C 58±10 | 6±5 | 2/upper arms | Number of steps (Digiwalker), energy expenditure (Senswear), manual step counting and indirect calorimetry | | No significant correlation with manual step counting; inconsistent correlation with calorimetry r=0.56-0.85; Test-retest for both sensors ICC=0.61-0.98 |
| Tieges 2015 [62] | 96 | Median 72  IQR 64-80 | 1,6 and 12 months | 1/unaffected leg | Commercial system (activPAL); sedentary time, clinical outcomes (NIHSS, NEADL questionnaire) | | Overall sedentary time per day 81%; Median bout time 1.7 hrs; No change in sedentary or bout time over time; Sedentary time was associated with NIHSS and NEADL, and bout time with age |
| ***Upper extremity activity, stroke*** | | | | | | | |
| Gebruers 2008 [40] | 39 | 74±10 | ≤ 7 days | 2/wrists | Actigraph with Java program; arm activity, ratio (impaired/unimpaired arm), clinical scales (NIHSS, FMA) | | Correlations: NIHSS: ratio r= -0.59, arm activity r= -0.57; FMA: ratio r= 0.54, arm activity r=0.69; NIHSS (cutoff 7): ratio: Sens 0.90, Spec 0.75; arm activity: Sens 0.84, Spec 0.75; FMA (cutoff 45): ratio: Sens 1.00, Spec 0.85; arm activity: Sens 0.95, Spec 0.85; |
| Gebruers 2013 [41] | 129  19 C | 74±11  C 71±14 | ≤7 days | 2/wrists | Actigraph with Java program; arm activity, ratio (impaired/unimpaired arm) | | Impaired arm activity: r=0.59 ratio; mRS r= -0.48; mRS (cutoff ≤2): Sen 0.80-0.85; Spec 0.75-0.77 |
| Le Heron 2014 [42] | 20  10 C | 77(59-82)  C 64(48-71) | 54 (47-100) hrs | 2/wrists | Algorithm (Machine learning, Fast Fourier transformations); NIHSS | | Correlation with NIHSS: r= -0.53; Sens 0.95; Spec 0.6; PPV 0.83; NPV 0.86 |
| Lang 2007 [44] | 34  10 C | 64±15  C 59±13 | 9±4 days | 2/wrists | Activity monitors; arm use in 24 hrs. clinical assessments (ARAT, WMFT) | | Affected arm 3.3±1.8 hrs, unaffected arm 6.0±4.6 hrs, less activity time than controls; AROM, strength and shoulder pain were correlated with affected arm use; Wrist extension and AROM had highest correlation r=0.63; Correlation with: ARAT0.40, WMFT function/time 0.62/-0.65, FIM 0.56-0.67 |
| Thrane 2011 [43] | 31 | 65 ± 14 | 11±6 days | 2/wrists | Commercial system (ActiGraph); FMA, self-care dependency, arm ratio, duration | | Correlation: FMA and affected arm use r= 0.60; FMA and arm ratio r=-0.85; Dependency was associated with arm ratio |
| de Niet 2007 [45] | 17  5 C | 54±14  C 43±13 | 33±22 months | 5/thighs, wrists, sternum; 2/elbows (goniometer) | Algorithm based; stroke-ULAM (accelerometry and electro-goniometry); arm use, proportion of use | | ULAM discriminated between different function levels; proportions of use 23-39% in stroke, 85-93% in controls |
| van der Pas 2011 [46] | 45 | 59±9 | 2±2 | 2/wrists | Commercial system (Actiwatch); MAL amount of use, MAL-26 quality of movement, SIS | | Correlation: MAL: affected arm r= 0.58, ratio r=0.60; MAL-26: ratio r= 0.66; SIS: ratio r=0.58; |
| Shim 2014 [47] | 40 | 59±15 | 15±4 months | 2/wrists | Commercial system (FITMETER); amount of arm activity ratio (affected/less-affected) | | Affected arm use and ratio better in well recovered than moderately recovered group (FMA cut off 45). No difference in unaffected arm |
| Uswatte 2005 [48] | 10 CIMT  10 non-CIMT | 61±20;  non-CIMT 64±14 | > 1 | 4/wrists, chest, more affected ankle | Commercial system; proportion of movement duration, ratio, MAL | | Test-retest reliability of more impaired, less impaired arm and leg r= 0.82-0.94; Improved ratio in intervention (CIMT) group; Correlation with MAL: r=0.74; |
| Uswatte 2006 [49] | 82  87 C | 63.0±12.8  C 64.2±12.7 | 3-9 months | 2/wrists | Commercial system; proportion of movement duration, ratio; clinical scales (MAL, AAUT) | | Correlation between two time periods: r= 0.81-0.90;  MAL: ratio r= 0.52, impaired arm r=0.41;  AAUT: ratio r= 0.60, impaired arm r=0.38; |
| Michielsen 2012 [50] | 38  18 C | 57±13  C 48±11 | 5±3 | 5/thighs, wrists, sternum | Commercial system (stroke-ULAM); duration of body postures and movements | | Use of unaffected arm(5.3 hrs), affected arm (2.4 hrs), controls (5.1-5.4hrs); Paretic side had lower intensity in bimanual activities; Time in standing/lying (2.1/10.8 hrs)in stroke, controls (3.6/ 8.9hrs); No difference in sitting time |
| Urbin 2015 [51] | 8 Inpat;  27 Outpat | Inpat 56±10  Outpat 62±9 | Inpat < 30 days; Outpat > 6 months | 2/wrists | Acceleration metrics sensitivity to change after task-specific training; ARAT | | 5 metrics improved: usage time, magnitude and variation ratio, median paretic arm acceleration magnitude and variability; Correlation with ARAT r=0.73-0.85; |
| ***Step counts, stroke*** | | | | | | | |
| Mudge 2008 [52] | 40 | 69.2 ± 12.6 | > 6 months | 1/ non-paretic ankle | Commercial system (SAM); Total step count, number of steps at high medium and low stepping rates | | Reliability and agreement: all outputs of SAM: ICC=0.83-0.99; Lowest agreement for one-day monitoring; LOA for 3-days: total step count ±37.8%; Highest step rate in 1 min ±23.0%; Highest step rate in 5 min ± 38.6% and peak activity index ±29.8% |
| Mudge 2007 [53] | 25 | 69 (42-79) | > 6 months | 2/ankles | Step count (SAM), 3D gait analysis and footswitches | | Correlation: 3D gait (indoors): non-paretic, r=0.959; paretic, r=0.896; LOA ±10 steps; Footswitches (outdoors): non-paretic, r=0.99, LOA ±9 steps, paretic, r=0.96, LOA±57 steps |
| Fulk 2014 [54] | 30  20 TBI | S 62 ±10  TBI 40±12 | 57±53 months  TBI 81 ±118 months | 2/wrist, waist (less affected side) | Step counts during 2 min walking test (SAM, FitBit Ultra, Pedometer, Nike+ Fuelband), video | | Agreement with video: SAM: ICC=0.97, mean difference 4.7; Fitbit Ultra: ICC=0.7, mean difference 9.7; Pedometer: ICC=0.42, mean difference 28.8; Nike+Fuelband ICC= 0.20, mean difference 66.2 |
| ***Walking activity, stroke*** | | | | | | | |
| Sanchez 2015 [60] | 23  20 C | 58±13;  C 55±13 | ≤ 4 days, 12 and 48 weeks | 3/thighs, sternum | Commercial system (Vitaport); Walking behavior parameters at 1, 12 and 48 weeks after stroke | | Responsiveness: no significant improvement in mean walking time; Time in walking, time upright; number of walking bouts increased during week 1 and 12; Step regularity, gait symmetry and gait speed increased consistently; Significant differences at 48 weeks in step regularity, gait speed compared to controls. |
| Prajapati 2011 [61] | 16 | 60 ±15 | 38±25 days | 2/ankles | Symmetry measures between (ABLE system), GAITRite; clinical scale (BBS) | | Mean walking time: 9.7% (8hrs); Mean duration of walking bouts: 54s; Larger asymmetry during free walking compared to GAITRite; correlation between speed and BBS r=0.60 |

*Sens* sensitivity*, Spec* specificity*, hrs* hours*, min* minutes*, Lab* laboratory*, FreeAct* free activities*, SD* standard deviation*, GTCS* generalized tonic-clonic seizures*, video-EEG* video electroencephalogram, *PNES* psychogenic non-epileptic seizures*, ES* epileptic seizures*, PPV* positive predictive value*, NPV* negative predictive value*, PD* Parkinson’s disease*, C* controls*, H&Y stage* Hoehn and Yahr stage*, UPDRS* Unified Parkinson’s Disease Rating Scale*, CDRS* Clinical Dyskinesia Rating Scale*, LID* Levodopa-Induced Dyskinesia, *mAIMS* modified Abnormal Involuntary Movement Scale*, UDysRS,* Unified Dyskinesia Rating Scale*, IQR* interquartile range*, ICC* intraclass correlation*, MBRS* modified Bradykinesia Rating Scale*, FOG* freezing of gait*, PIGD* postural instability and gait disorder subscore*, SAM* StepWatch Activity Monitor*, MiniBESTest* Mini Balance Evaluation Systems Test*, BBS* Berg Balance Scale*, NIHSS* the Nation Institutes of Health Stroke Scale*, NEADL* the Nottingham Extended Activities of Daily Living Questionnaire*, LOA* limits of agreement*, 6MWT* 6-minutes walking test*, FMA* Fugl-Meyer Assessment*, AROM* active ranges of motion*, ARAT* the Action Research Arm Test*, WMFT* the Wolf Motor Function Test*, FIM* the Functional Independence Measure*, MAL* The Motor Activity Log*, CIMT* Consecutive constraint-Induced Movement therapy*, AAUT* Actual Amount of Use Test*, mRS* modified Rankin Scale
